# The genome sequence of the Ibiza wall lizard, *Podarcis pityusensis *(Boscá, 1883)

**DOI:** 10.12688/wellcomeopenres.24143.1

**Published:** 2025-05-07

**Authors:** Nathalie Feiner, Tobias Uller, Ferran de la Cruz, Roberto Garcia-Roa, Joana Meier, Wellcome Sanger Institute Tree of Life Management, Samples and Laboratory team, Wellcome Sanger Institute Scientific Operations: Sequencing Operations, Wellcome Sanger Institute Tree of Life Core Informatics team, Tree of Life Core Informatics collective

**Affiliations:** 1 Max Planck Institute for Evolutionary Biology, Plön, Germany; 2 Department of Biology, Lund University, Lund, Sweden; 3 CIBIO Research Centre in Biodiversity and Genetic Resources, InBIO Associate Laboratory, Universidade do Porto, Porto, Portugal; 4 BIOPOLIS Program in Genomics, Biodiversity and Land Planning, CIBIO, Campus de Vairão, Vairão, Portugal; 5 Departamento de Biologia, Universidade do Porto Faculdade de Ciencias, Porto, Portugal; 6 Tree of Life, Wellcome Sanger Institute, Hinxton, England, UK

**Keywords:** Podarcis pityusensis, Ibiza wall lizard, genome sequence, chromosomal, Squamata

## Abstract

Eukaryota; Opisthokonta; Metazoa; Eumetazoa; Bilateria; Deuterostomia; Chordata; Craniata; Vertebrata; Gnathostomata; Teleostomi; Euteleostomi; Sarcopterygii; Dipnotetrapodomorpha; Tetrapoda; Amniota; Sauropsida; Sauria; Lepidosauria; Squamata; Bifurcata; Unidentata; Episquamata; Laterata; Lacertibaenia; Lacertidae; Lacertinae; *Podarcis*; *Podarcis pityusensis* (Boscá, 1883) (NCBI:txid74359)

## Species taxonomy

Eukaryota; Opisthokonta; Metazoa; Eumetazoa; Bilateria; Deuterostomia; Chordata; Craniata; Vertebrata; Gnathostomata; Teleostomi; Euteleostomi; Sarcopterygii; Dipnotetrapodomorpha; Tetrapoda; Amniota; Sauropsida; Sauria; Lepidosauria; Squamata; Bifurcata; Unidentata; Episquamata; Laterata; Lacertibaenia; Lacertidae; Lacertinae; *Podarcis*; *Podarcis pityusensis* (Boscá, 1883) (NCBI:txid74359)

## Background

**Figure 1.**
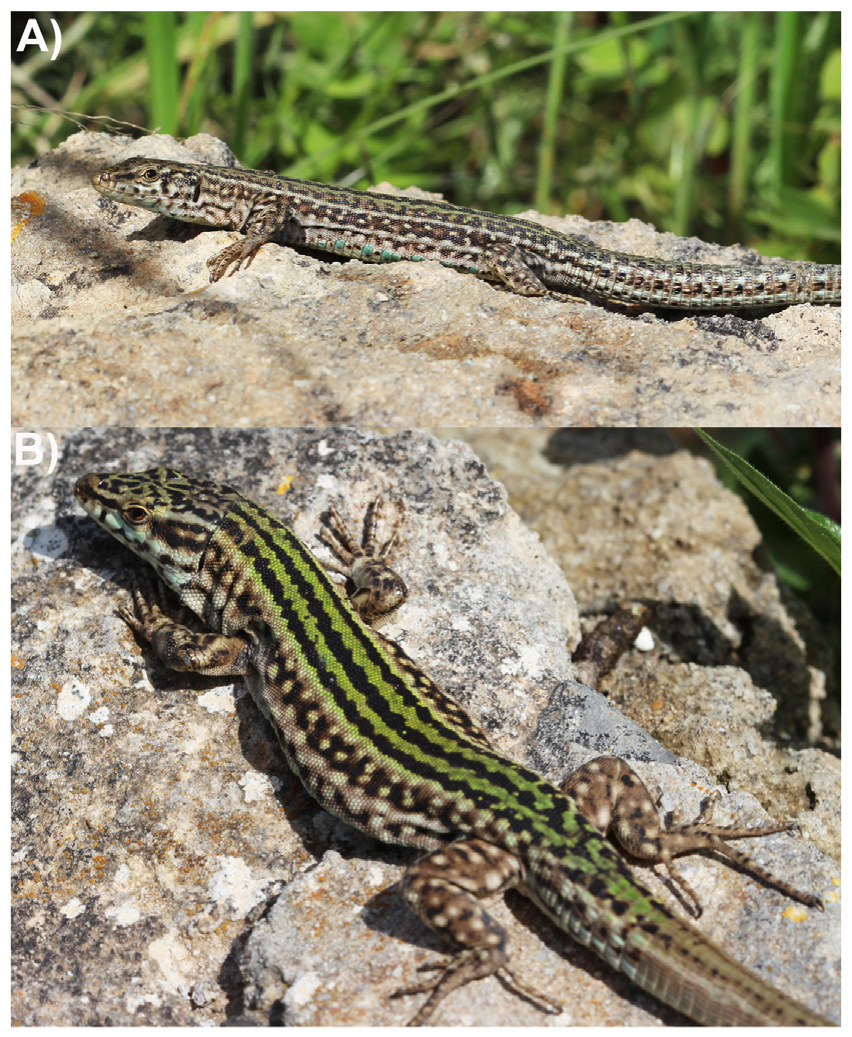
**A**) Female and **B**) male *Podarcis pityusensis* photographed in Cala Vedella (April 2022). Note that the specimen selected for genome sequencing is from the same locality, but not shown on the photographs. Photographs by Ferran de la Cruz.

The Ibiza wall lizard *Podarcis pityusensis* (Boscá, 1883; Figure 1) is a diurnal lacertid lizard endemic to Ibiza, Formentera and surrounding islets. More than 20 subspecies have been described, reflecting the large phenotypic diversity found in this species ( Böhme, 1986). Introduced populations of *P. pityusensis* are present in Northern and Eastern Spain and on Mallorca ( Colodro *et al.*, 2020; Zawadzki, 2020). As a consequence of its restricted distribution and recent decline on Ibiza, the species is classified as Endangered (EN) by the IUCN ( Bowles, 2024). Populations on the main island of Ibiza are particularly threatened by the invasive horseshoe whip snakes ( *Hemorrhois hippocrepis*) that were introduced with ornamental plants ( Montes *et al.*, 2022). Reports suggest that these snakes may even be capable of reaching nearby islets, potentially eradicating islet populations of *P. pityusensis* ( Montes *et al.*, 2022). The species has also suffered from the pet trade and is listed in Annex II of the Convention on International Trade in Endangered Species of Wild Fauna and Flora ( Zawadzki, 2020).

*Podarcis pityusensis* is one of 28 currently described species of the genus *Podarcis*. It is the sister species to *P. lilfordi* ( Yang *et al.*, 2021), which is another endemic to the Balearic islands inhabiting the Cabrera archipelago and islets surrounding Mallorca and Menorca. The two species likely separated following the post-Messinian rise in sea levels ( Brown *et al.*, 2008).

The Ibiza wall lizard *P. pityusensis* occurs in a variety of habitats, including Mediterranean shrubland, shrubby vegetation, grassland, sandy or rocky shores as well as urbanized environments. The population densities can be very high and lizards can vary substantially across islands in aspects of their biology, such as behaviour, colouration and body size ( Cooper & Pérez-Mellado, 2012). Colour variation includes reticulated, striped or plain patterns in blue, grey, black, brown, green and yellow hues. The main activity period is in spring and early summer when reproduction occurs, but lizards can be active throughout the year. The species is oviparous and Meiri *et al.* (2020) report an average clutch size of 2.65.

*P. pityusensis* is one of three species of the Western Islands group of *Podarcis*. This group is a descendant of a lineage formed through extensive introgressive hybridisation between lizards belonging to the eastern and western clade of *Podarcis* ( Yang *et al.*, 2021). Reference genomes of its sister species *P. tiliguerta* and *P. lilfordi* are available ( Gomez-Garrido *et al.*, 2023). The availability of reference genomes for all three species in this group will be a valuable resource for comparative studies above the species level. We anticipate that the reference genome of *P. pityusensis* will support ongoing conservation efforts and facilitate further studies of population history, gene flow, and causes of phenotypic diversification within this species.

## Genome sequence report

### Sequencing data

The genome of a specimen of *Podarcis pityusensis* was sequenced using Pacific Biosciences single-molecule HiFi long reads, generating 35.44 Gb (gigabases) from 5.36 million reads. GenomeScope analysis of the PacBio HiFi data estimated the haploid genome size at 1,461.54 Mb, with a heterozygosity of 0.57% and repeat content of 20.66%. These values provide an initial assessment of genome complexity and the challenges anticipated during assembly. Based on this estimated genome size, the sequencing data provided approximately 23.0x coverage of the genome. Chromosome conformation Hi-C sequencing produced 555.30 Gb from 3,677.49 million reads. Table 1 summarises the specimen and sequencing information.

**Table 1.** Specimen and sequencing data for *Podarcis pityusensis*.

Project information			
**Study title**	Podarcis pityusensis (Ibiza wall lizard)		
**Umbrella BioProject**	PRJEB73697		
**Species**	*Podarcis pityusensis*		
**BioSpecimen**	SAMEA114217797		
**NCBI taxonomy ID**	74359		
Specimen information			
**Technology**	**ToLID**	**BioSample accession**	**Organism part**
**PacBio long read sequencing**	rPodPit1	SAMEA114217801	terminal body
**Hi-C sequencing**	rPodPit1	SAMEA114217801	terminal body
Sequencing information			
**Platform**	**Run accession**	**Read count**	**Base count (Gb)**
**Hi-C Illumina NovaSeq X**	ERR12743668	3.68e+09	555.3
**PacBio Revio**	ERR12736921	5.36e+06	35.44

### Assembly statistics

The genome was assembled into two haplotypes using Hi-C phasing. Haplotype 1 was curated to chromosome level, while haplotype 2 was assembled to scaffold level. The assembly was improved by manual curation, which corrected 216 misjoins or missing joins and removed 9 haplotypic duplications. These interventions increased the total assembly length by 1.31%, decreased the scaffold count by 11.58%, and increased the scaffold N50 by 0.63%. The final assembly has a total length of 1,515.36 Mb in 335 scaffolds, with 1,506 gaps, and a scaffold N50 of 91.58 Mb ( Table 2).

**Table 2.** Genome assembly data for *Podarcis pityusensis*.

Genome assembly	Haplotype 1	Haplotype 2
Assembly name	rPodPit1.hap1.2	rPodPit1.hap2.2
Assembly accession	GCA_964106645.2	GCA_964106635.2
Assembly level	chromosome	chromosome
Span (Mb)	1,515.36	1,417.08
Number of contigs	1,841	1,548
Number of scaffolds	335	183
Longest scaffold (Mb)	139.68	139.78
Assembly metrics (benchmark)	Haplotype 1	Haplotype 2
Contig N50 length (≥ 1 Mb)	1.61 Mb	1.72 Mb
Scaffold N50 length (= chromosome N50)	91.58 Mb	92.42 Mb
Consensus quality (QV) (≥ 40)	60.6	60.8
*k*-mer completeness	86.42%	82.68%
Combined *k*-mer completeness (≥ 95%)	97.94%	
BUSCO * (S > 90%; D < 5%)	C:94.9%[S:92.7%,D:2.2%], F:0.9%,M:4.2%,n:7480	-
Percentage of assembly mapped to chromosomes (≥ 90%)	99.0%	99.43%
Sex chromosomes (localised homologous pairs)	W and Z	-
Organelles (one complete allele)	Mitochondrial genome: 17.19 kb	-

* BUSCO scores based on the sauropsida_odb10 BUSCO set using version 5.5.0. C = complete [S = single copy, D = duplicated], F = fragmented, M = missing, n = number of orthologues in comparison.

**Figure 2.**
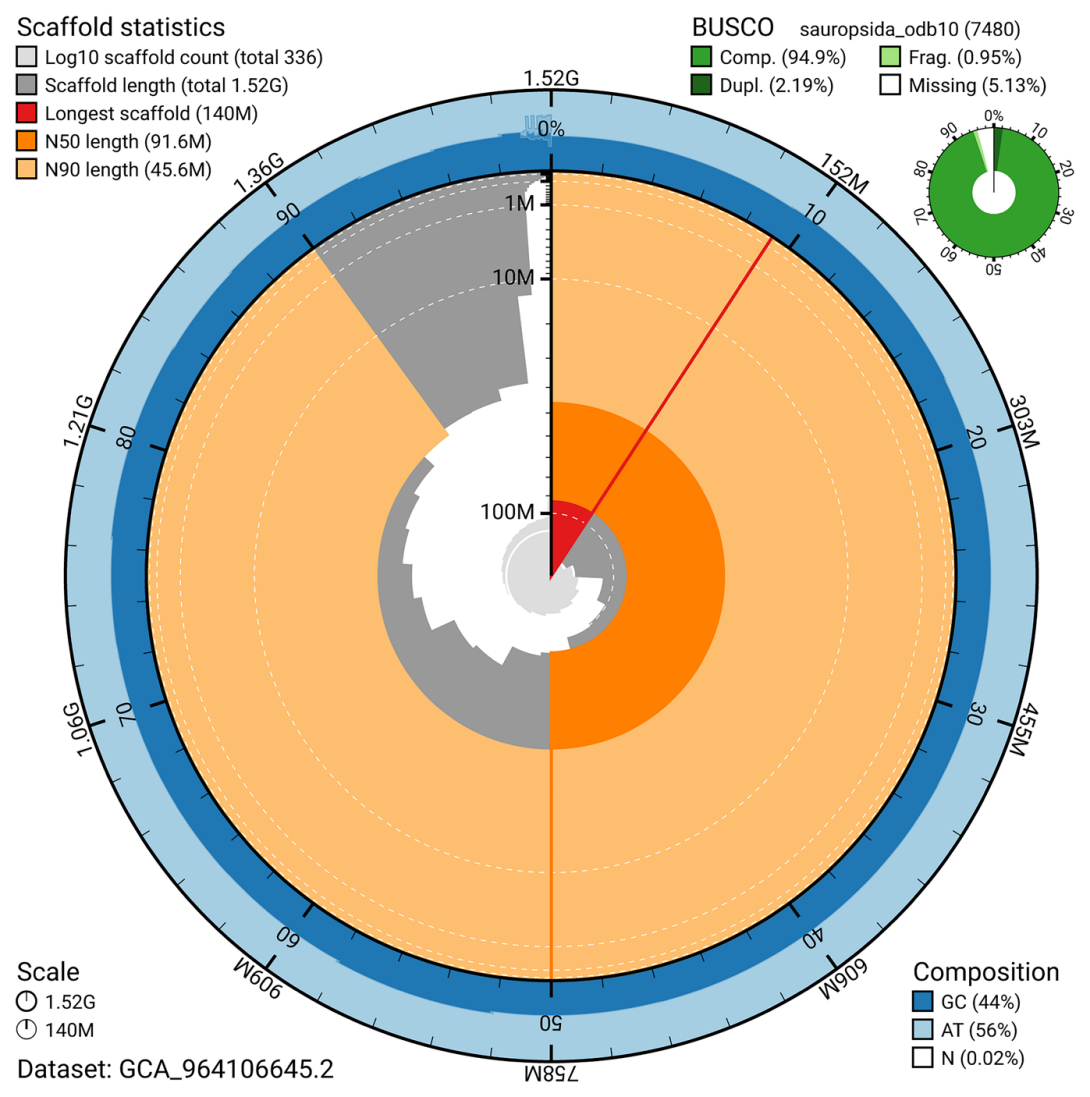
Genome assembly of *Podarcis pityusensis*, rPodPit1.hap1.2: metrics. The BlobToolKit snail plot provides an overview of assembly metrics and BUSCO gene completeness. The circumference represents the length of the whole genome sequence, and the main plot is divided into 1,000 bins around the circumference. The outermost blue tracks display the distribution of GC, AT, and N percentages across the bins. Scaffolds are arranged clockwise from longest to shortest and are depicted in dark grey. The longest scaffold is indicated by the red arc, and the deeper orange and pale orange arcs represent the N50 and N90 lengths. A light grey spiral at the centre shows the cumulative scaffold count on a logarithmic scale. A summary of complete, fragmented, duplicated, and missing BUSCO genes in the sauropsida_odb10 set is presented at the top right. An interactive version of this figure is available at https://blobtoolkit.genomehubs.org/view/GCA_964106645.2/dataset/GCA_964106645.2/snail.

**Figure 3.**
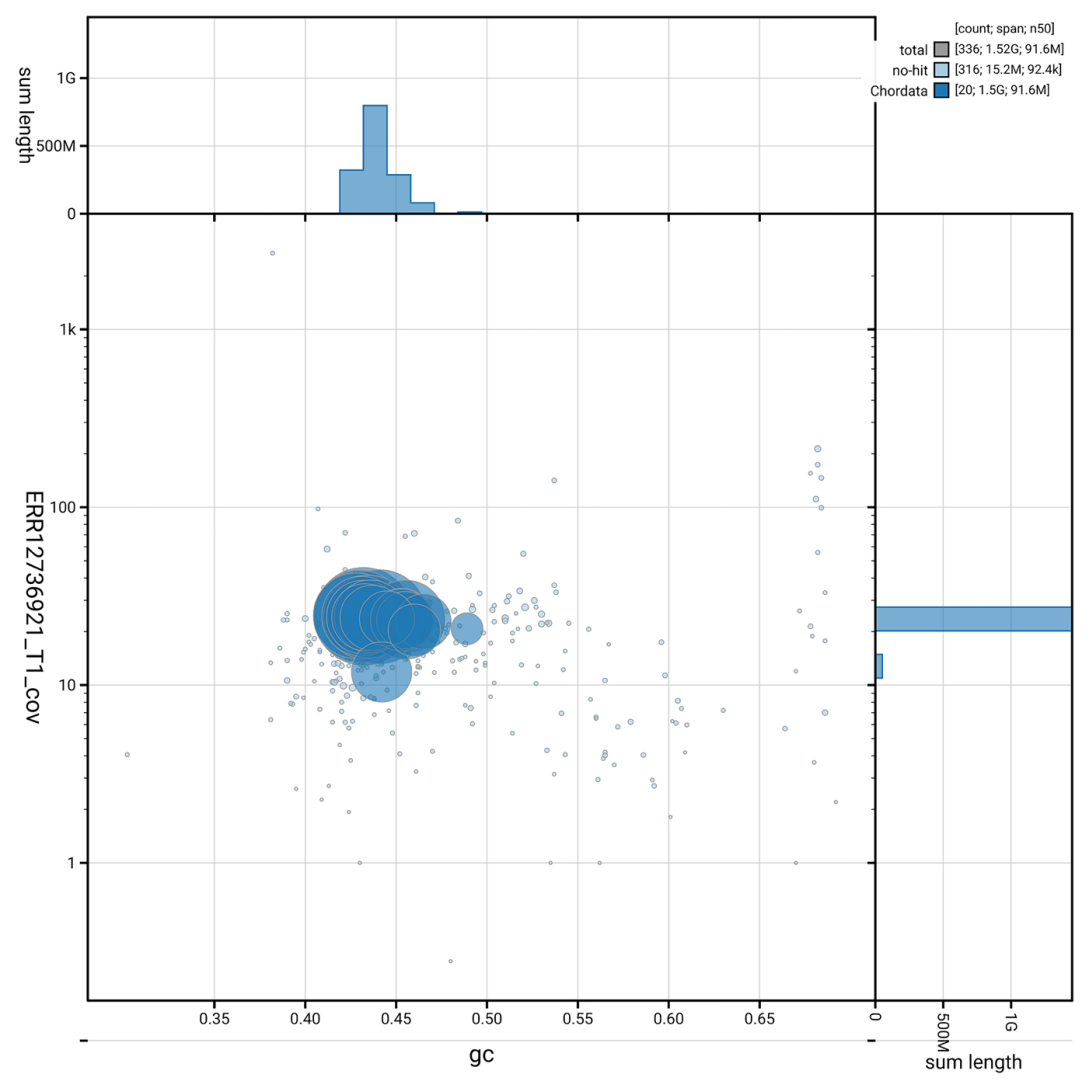
Genome assembly of *Podarcis pityusensis*, rPodPit1.hap1.2: BlobToolKit GC-coverage plot. Blob plot showing sequence coverage (vertical axis) and GC content (horizontal axis). The circles represent scaffolds, with the size proportional to scaffold length and the colour representing phylum membership. The histograms along the axes display the total length of sequences distributed across different levels of coverage and GC content. An interactive version of this figure is available at https://blobtoolkit.genomehubs.org/view/GCA_964106645.2/dataset/GCA_964106645.2/blob.

**Figure 4.**
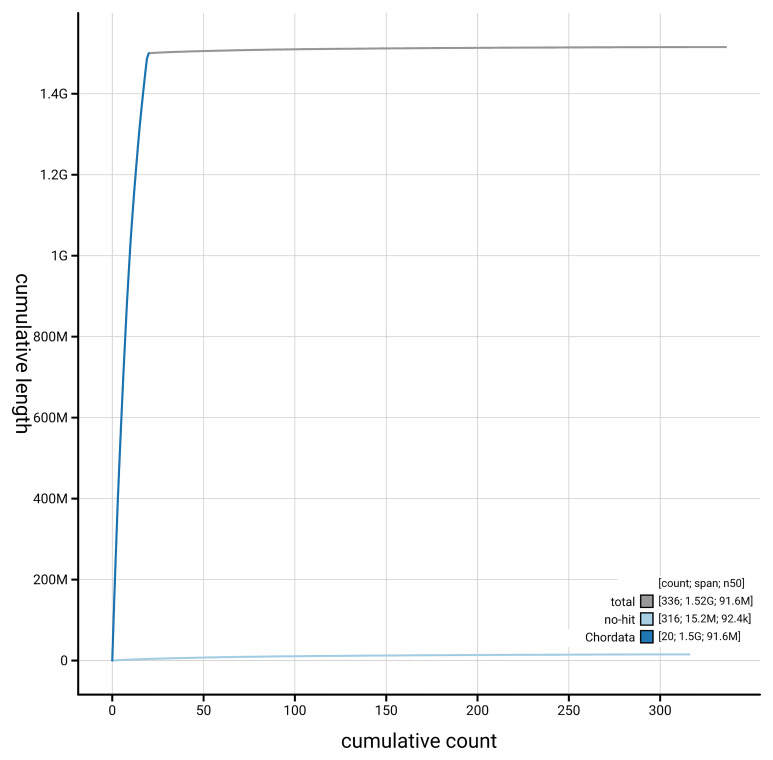
Genome assembly of *Podarcis pityusensis*, rPodPit1.hap1.2: BlobToolKit cumulative sequence plot. The grey line shows cumulative length for all scaffolds. Coloured lines show cumulative lengths of scaffolds assigned to each phylum using the buscogenes taxrule. An interactive version of this figure is available at https://blobtoolkit.genomehubs.org/view/GCA_964106645.2/dataset/GCA_964106645.2/cumulative.

The snail plot in Figure 2 provides a summary of the assembly statistics, indicating the distribution of scaffold lengths and other assembly metrics. Figure 3 shows the distribution of scaffolds by GC proportion and coverage. Figure 4 presents a cumulative assembly plot, with separate curves representing different scaffold subsets assigned to various phyla, illustrating the completeness of the assembly.

**Figure 5.**
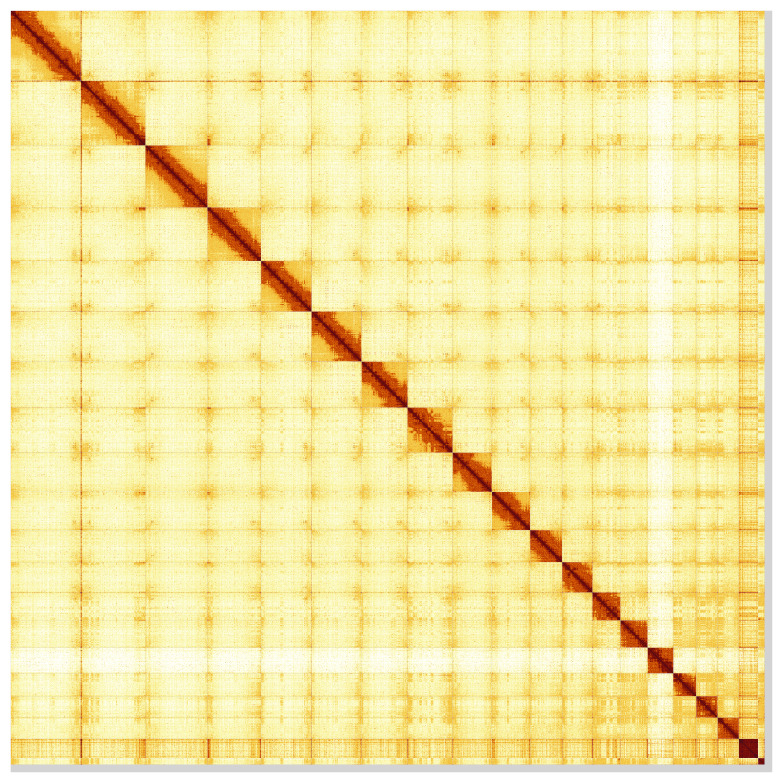
Genome assembly of *Podarcis pityusensis*: Hi-C contact map of the rPodPit1.hap1.2 assembly, visualised using HiGlass. Chromosomes are shown in order of size from left to right and top to bottom. An interactive version of this figure may be viewed at https://genome-note-higlass.tol.sanger.ac.uk/l/?d=X5Qm-ZHpRBSosl1GZmTKWA.

Most of the assembly sequence (99.0%) was assigned to 20 chromosomal-level scaffolds, representing 18 autosomes and the W and Z sex chromosomes. These chromosome-level scaffolds, confirmed by Hi-C data, are named according to size ( Figure 5; Table 3). During curation, the sex chromosomes were assigned by synteny to the genome of *Podarcis raffonei* (GCA_027172205.1) and by PacBio reads coverage observed in the Hi-C map.

**Table 3.** Chromosomal pseudomolecules in the genome assembly of *Podarcis pityusensis*, rPodPit1.

Haplotype 1				Haplotype 2			
INSDC accession	Name	Length (Mb)	GC%	INSDC accession	Name	Length (Mb)	GC%
OZ067038.1	1	139.68	43	OZ067003.1	1	139.78	43
OZ067039.1	2	128.38	44	OZ067004.1	2	126.52	44
OZ067040.1	3	123.67	43	OZ067005.1	3	122.66	43
OZ067041.1	4	106.34	43	OZ067006.1	4	107.44	43
OZ067042.1	5	100.81	43.5	OZ067007.1	5	100.53	43
OZ067043.1	6	99.26	43.5	OZ067008.1	6	99.09	43.5
OZ067044.1	7	91.58	43	OZ067009.1	7	92.42	43
OZ067045.1	8	89.68	45.5	OZ067010.1	8	89.26	45.5
OZ067046.1	9	78.23	43	OZ067011.1	9	78.36	43
OZ067047.1	10	75.91	43.5	OZ067012.1	10	76.05	43.5
OZ067048.1	11	63.86	43.5	OZ067013.1	11	64.48	43.5
OZ067049.1	12	60.19	43.5	OZ067014.1	12	60.35	43.5
OZ067050.1	13	55.79	45.5	OZ067015.1	13	55.12	45.5
OZ067051.1	14	54.0	45.5	OZ067016.1	14	53.75	45.5
OZ067052.1	15	45.56	45.5	OZ067017.1	15	45.55	45.5
OZ067053.1	16	42.64	46.5	OZ067018.1	16	42.68	46.5
OZ067054.1	17	42.41	44.5	OZ067019.1	17	42.29	44.5
OZ067055.1	18	13.05	49	OZ067020.1	18	12.74	49
OZ078260.1	W	38.07	46				
OZ078259.1	Z	51.1	44				
OZ067056.1	MT	0.02	39				

The mitochondrial genome was also assembled. This sequence is included as a contig in the multifasta file of the genome submission and as a standalone record.

### Assembly quality metrics

The estimated Quality Value (QV) and *k*-mer completeness metrics, along with BUSCO completeness scores, were calculated for each haplotype and the combined assembly. The QV reflects the base-level accuracy of the assembly, while *k*-mer completeness indicates the proportion of expected *k*-mers identified in the assembly. BUSCO scores provide a measure of completeness based on benchmarking universal single-copy orthologues.

For haplotype 1, the estimated QV is 60.6, and for haplotype 2, 60.8. When the two haplotypes are combined, the assembly achieves an estimated QV of 60.7. The *k*-mer recovery for haplotype 1 is 86.42%, and for haplotype 2 82.68%, while the combined haplotypes have a *k*-mer recovery of 97.94%. BUSCO 5.5.0 analysis using the sauropsida_odb10 reference set ( *n* = 7,480) identified 94.9% of the expected gene set (single = 92.7%, duplicated = 2.2%) for haplotype 1.

Table 2 provides assembly metric benchmarks adapted from Rhie *et al.* (2021) and the Earth BioGenome Project (EBP) Report on Assembly Standards September 2024. The assembly achieves the EBP reference standard of **6.C.Q60.**

## Methods

### Sample acquisition

The specimen, an adult female *P. pityusensis* lizard (specimen ID SAN25001763, ToLID rPodPit1) was collected on 2022-04-19 from a site near Cala Vedella on the main island of Ibiza (latitude: 38.9105995; longitude: 1.2240528). The specimen was caught by noosing, standard morphometric measurements were taken and the tip of the tail (ca. 2 cm) was collected and preserved in ethanol. The specimen was released again at the site of capture. Field work was conducted under the permit ID CEP 03/2022. The specimen was collected and identified by Roberto García-Roa (University of Lund, Sweden).

### Nucleic acid extraction

The workflow for high molecular weight (HMW) DNA extraction at the Wellcome Sanger Institute (WSI) Tree of Life Core Laboratory includes a sequence of procedures: sample preparation and homogenisation, DNA extraction, fragmentation and purification. Detailed protocols are available on protocols.io ( Denton *et al.*, 2023b). The rPodPit1 sample was prepared for DNA extraction by weighing and dissecting it on dry ice ( Jay *et al.*, 2023). Tissue from the terminal body was homogenised using a PowerMasher II tissue disruptor ( Denton *et al.*, 2023a). HMW DNA was extracted using the Manual MagAttract v1 protocol ( Strickland *et al.*, 2023b). DNA was sheared into an average fragment size of 12–20 kb in a Megaruptor 3 system ( Todorovic *et al.*, 2023). Sheared DNA was purified by solid-phase reversible immobilisation, using AMPure PB beads to eliminate shorter fragments and concentrate the DNA ( Strickland *et al.*, 2023a). The concentration of the sheared and purified DNA was assessed using a Nanodrop spectrophotometer and Qubit Fluorometer using the Qubit dsDNA High Sensitivity Assay kit. Fragment size distribution was evaluated by running the sample on the FemtoPulse system.

### Hi-C sample preparation

Hi-C data were generated from the terminal body of the rPodPit1 sample using the Arima-HiC v2 kit (Arima Genomics) with 20–50 mg of frozen tissue (stored at –80 °C). As per manufacturer’s instructions, tissue was fixed, and the DNA crosslinked using a TC buffer with a final formaldehyde concentration of 2%. The tissue was then homogenised using the Diagnocine Power Masher-II. The crosslinked DNA was digested using a restriction enzyme master mix, then biotinylated and ligated. A clean up was performed with SPRIselect beads prior to library preparation. DNA concentration was quantified using the Qubit Fluorometer v4.0 (Thermo Fisher Scientific) and Qubit HS Assay Kit, and sample biotinylation percentage was estimated using the Arima-HiC v2 QC beads.

### Library preparation and sequencing

Library preparation and sequencing were performed at the WSI Scientific Operations core.

***PacBio HiFi***

At a minimum, samples were required to have an average fragment size exceeding 8 kb and a total mass over 400 ng to proceed to the low input SMRTbell Prep Kit 3.0 protocol (Pacific Biosciences, California, USA), depending on genome size and sequencing depth required. Libraries were prepared using the SMRTbell Prep Kit 3.0 (Pacific Biosciences, California, USA) as per the manufacturer's instructions. The kit includes the reagents required for end repair/A-tailing, adapter ligation, post-ligation SMRTbell bead cleanup, and nuclease treatment. Following the manufacturer’s instructions, size selection and clean up was carried out using diluted AMPure PB beads (Pacific Biosciences, California, USA). DNA concentration was quantified using the Qubit Fluorometer v4.0 (Thermo Fisher Scientific) with Qubit 1X dsDNA HS assay kit and the final library fragment size analysis was carried out using the Agilent Femto Pulse Automated Pulsed Field CE Instrument (Agilent Technologies) and gDNA 55kb BAC analysis kit.

Samples were sequenced on a Revio instrument (Pacific Biosciences, California, USA). Prepared libraries were normalised to 2 nM, and 15 μL was used for making complexes. Primers were annealed and polymerases were hybridised to create circularised complexes according to manufacturer’s instructions. The complexes were purified with the 1.2X clean up with SMRTbell beads. The purified complexes were then diluted to the Revio loading concentration (in the range 200–300 pM), and spiked with a Revio sequencing internal control. Samples were sequenced on Revio 25M SMRT cells (Pacific Biosciences, California, USA). The SMRT link software, a PacBio web-based end-to-end workflow manager, was used to set-up and monitor the run, as well as perform primary and secondary analysis of the data upon completion.

***Hi-C***

For Hi-C library preparation, the biotinylated DNA constructs were fragmented using a Covaris E220 sonicator and size-selected to 400–600 bp using SPRISelect beads. DNA was then enriched using Arima-HiC v2 Enrichment beads. The NEBNext Ultra II DNA Library Prep Kit (New England Biolabs) was used for end repair, A-tailing, and adapter ligation, following a modified protocol in which library preparation is carried out while the DNA remains bound to the enrichment beads. PCR amplification was performed using KAPA HiFi HotStart mix and custom dual-indexed adapters (Integrated DNA Technologies) in a 96-well plate format. Depending on sample concentration and biotinylation percentage determined at the crosslinking stage, samples were amplified for 10–16 PCR cycles. Post-PCR clean-up was carried out using SPRISelect beads. The libraries were quantified using the Accuclear Ultra High Sensitivity dsDNA Standards Assay kit (Biotium) and normalised to 10 ng/μL before sequencing. Hi-C sequencing was performed on the Illumina NovaSeq X instrument using 150 bp paired-end reads.

### Genome assembly, curation and evaluation

***Assembly***

Prior to assembly of the PacBio HiFi reads, a database of *k*-mer counts ( *k* = 31) was generated from the filtered reads using FastK. GenomeScope2 ( Ranallo-Benavidez *et al.*, 2020) was used to analyse the *k*-mer frequency distributions, providing estimates of genome size, heterozygosity, and repeat content.

The HiFi reads were assembled using Hifiasm in Hi-C phasing mode ( Cheng *et al.*, 2021; Cheng *et al.*, 2022), resulting in a pair of haplotype-resolved assemblies. The Hi-C reads were mapped to the primary contigs using bwa-mem2 ( Vasimuddin *et al.*, 2019). The contigs were further scaffolded using the provided Hi-C data ( Rao *et al.*, 2014) in YaHS ( Zhou *et al.*, 2023) using the --break option for handling potential misassemblies. The scaffolded assemblies were evaluated using Gfastats ( Formenti *et al.*, 2022), BUSCO ( Manni *et al.*, 2021) and MERQURY.FK ( Rhie *et al.*, 2020).

The mitochondrial genome was assembled using MitoHiFi ( Uliano-Silva *et al.*, 2023), which runs MitoFinder ( Allio *et al.*, 2020) and uses these annotations to select the final mitochondrial contig and to ensure the general quality of the sequence.

***Assembly curation***

The assembly was decontaminated using the Assembly Screen for Cobionts and Contaminants (ASCC) pipeline. Flat files and maps used in curation were generated via the TreeVal pipeline ( Pointon *et al.*, 2023). Manual curation was conducted primarily in PretextView ( Harry, 2022) and HiGlass ( Kerpedjiev *et al.*, 2018), with additional insights provided by JBrowse2 ( Diesh *et al.*, 2023). Scaffolds were visually inspected and corrected as described by Howe *et al*. (2021). Any identified contamination, missed joins, and mis-joins were amended, and duplicate sequences were tagged and removed. The curation process is documented at https://gitlab.com/wtsi-grit/rapid-curation.

***Assembly quality assessment***

The Merqury.FK tool ( Rhie *et al.*, 2020), run in a Singularity container ( Kurtzer *et al.*, 2017), was used to evaluate *k*-mer completeness and assembly quality for both haplotypes using the *k*-mer databases ( *k* = 31) computed prior to genome assembly. The analysis outputs included assembly QV scores and completeness statistics.

A Hi-C contact map was produced for the final version of the assembly. The Hi-C reads were aligned using bwa-mem2 ( Vasimuddin *et al.*, 2019) and the alignment files were combined using SAMtools ( Danecek *et al.*, 2021). The Hi-C alignments were converted into a contact map using BEDTools ( Quinlan & Hall, 2010) and the Cooler tool suite ( Abdennur & Mirny, 2020). The contact map was visualised in HiGlass ( Kerpedjiev *et al.*, 2018).

The blobtoolkit pipeline is a Nextflow ( Di Tommaso *et al.*, 2017) port of the previous Snakemake Blobtoolkit pipeline ( Challis *et al.*, 2020). It aligns the PacBio reads in SAMtools and minimap2 ( Li, 2018) and generates coverage tracks for regions of fixed size. In parallel, it queries the GoaT database ( Challis *et al.*, 2023) to identify all matching BUSCO lineages to run BUSCO ( Manni *et al.*, 2021). For the three domain-level BUSCO lineages, the pipeline aligns the BUSCO genes to the UniProt Reference Proteomes database ( Bateman *et al.*, 2023) with DIAMOND blastp ( Buchfink *et al.*, 2021). The genome is also divided into chunks according to the density of the BUSCO genes from the closest taxonomic lineage, and each chunk is aligned to the UniProt Reference Proteomes database using DIAMOND blastx. Genome sequences without a hit are chunked using seqtk and aligned to the NT database with blastn ( Altschul *et al.*, 1990). The blobtools suite combines all these outputs into a blobdir for visualisation.

The blobtoolkit pipeline was developed using nf-core tooling ( Ewels *et al.*, 2020) and MultiQC ( Ewels *et al.*, 2016), relying on the Conda package manager, the Bioconda initiative ( Grüning *et al.*, 2018), the Biocontainers infrastructure ( da Veiga Leprevost *et al.*, 2017), as well as the Docker ( Merkel, 2014) and Singularity ( Kurtzer *et al.*, 2017) containerisation solutions.

Table 4 contains a list of relevant software tool versions and sources.

**Table 4.** Software tools: versions and sources.

Software tool	Version	Source
BEDTools	2.30.0	https://github.com/arq5x/bedtools2
BLAST	2.14.0	ftp://ftp.ncbi.nlm.nih.gov/blast/executables/blast+/
BlobToolKit	4.3.9	https://github.com/blobtoolkit/blobtoolkit
BUSCO	5.5.0	https://gitlab.com/ezlab/busco
bwa-mem2	2.2.1	https://github.com/bwa-mem2/bwa-mem2
Cooler	0.8.11	https://github.com/open2c/cooler
DIAMOND	2.1.8	https://github.com/bbuchfink/diamond
fasta_windows	0.2.4	https://github.com/tolkit/fasta_windows
FastK	666652151335353eef2fcd58880bcef5bc2928e1	https://github.com/thegenemyers/FASTK
Gfastats	1.3.6	https://github.com/vgl-hub/gfastats
GoaT CLI	0.2.5	https://github.com/genomehubs/goat-cli
Hifiasm	0.19.8-r603	https://github.com/chhylp123/hifiasm
HiGlass	44086069ee7d4d3f6f3f0012569789ec138f42b84aa443578 26c0b6753eb28de	https://github.com/higlass/higlass
MerquryFK	d00d98157618f4e8d1a9190026b19b471055b22e	https://github.com/thegenemyers/MERQURY.FK
Minimap2	2.24-r1122	https://github.com/lh3/minimap2
MitoHiFi	3	https://github.com/marcelauliano/MitoHiFi
MultiQC	1.14, 1.17, and 1.18	https://github.com/MultiQC/MultiQC
NCBI Datasets	15.12.0	https://github.com/ncbi/datasets
Nextflow	23.10.0	https://github.com/nextflow-io/nextflow
PretextView	0.2.5	https://github.com/sanger-tol/PretextView
samtools	1.19.2	https://github.com/samtools/samtools
sanger-tol/ ascc	-	https://github.com/sanger-tol/ascc
sanger-tol/blobtoolkit	0.5.1	https://github.com/sanger-tol/blobtoolkit
Seqtk	1.3	https://github.com/lh3/seqtk
Singularity	3.9.0	https://github.com/sylabs/singularity
TreeVal	1.2.0	https://github.com/sanger-tol/treeval
YaHS	1.2a.2	https://github.com/c-zhou/yahs

### Wellcome Sanger Institute – Legal and Governance

The materials that have contributed to this genome note have been supplied by a Tree of Life collaborator. The Wellcome Sanger Institute employs a process whereby due diligence is carried out proportionate to the nature of the materials themselves, and the circumstances under which they have been/are to be collected and provided for use. The purpose of this is to address and mitigate any potential legal and/or ethical implications of receipt and use of the materials as part of the research project, and to ensure that in doing so we align with best practice wherever possible.

The overarching areas of consideration are:

•   Ethical review of provenance and sourcing of the material

•   Legality of collection, transfer and use (national and international)

Each transfer of samples is undertaken according to a Research Collaboration Agreement or Material Transfer Agreement entered into by the Tree of Life collaborator, Genome Research Limited (operating as the Wellcome Sanger Institute) and in some circumstances other Tree of Life collaborators.

## Data Availability

European Nucleotide Archive: *Podarcis pityusensis* (Ibiza wall lizard). Accession number PRJEB73697; https://identifiers.org/ena.embl/PRJEB73697. The genome sequence is released openly for reuse. The *Podarcis pityusensis* genome assembly is provided by the Wellcome Sanger Institute Tree of Life Programme ( https://www.sanger.ac.uk/programme/tree-of-life/). All raw sequence data and the assembly have been deposited in INSDC databases. The genome will be annotated using available RNA-Seq data and presented through the Ensembl pipeline at the European Bioinformatics Institute. Raw data and assembly accession identifiers are reported in Table 1 and Table 2.

## Author information

Members of the Wellcome Sanger Institute Tree of Life Management, Samples and Laboratory team are listed here: https://doi.org/10.5281/zenodo.12162482.

Members of Wellcome Sanger Institute Scientific Operations: Sequencing Operations are listed here: https://doi.org/10.5281/zenodo.12165051.

Members of the Wellcome Sanger Institute Tree of Life Core Informatics team are listed here: https://doi.org/10.5281/zenodo.12160324.

Members of the Tree of Life Core Informatics collective are listed here: https://doi.org/10.5281/zenodo.12205391.
