# Supplementary material for: The genome sequence of the Ibiza wall lizard, Podarcis pityusensis (Boscá, 1883)
Source: Wellcome Open Res. 2025 May 7;10:235. [Version 1] doi: 10.12688/wellcomeopenres.24143.1 (PMC12163369; doi:10.12688/wellcomeopenres.24143.1)
Supplement: Peer review history

# Reviewer report: Reviewer response for version 1

**DOI:** 10.21956/wellcomeopenres.26634.r123795

**Published:** 2025-06-12

**Authors:** Jessica Gomez-Garrido

**Affiliations:** 1 CNAG, Centro Nacional de Análisis Genómico, 08028, Barcelona, Barcelona, Spain

This genome note presents the genome assembly of Podarcis pityusensis, a species of the genus Podarcis that inhabits in Ibiza, Formentera and close islets. It is classified as endangered by the IUCN and the availability of its genome will be a key resource to support conservation efforts. 

 The sequencing and assembly strategies are appropriate, and the results are good. 2 haplotypes are provided, the first one containing both sex chromosomes and the mitogenome. Both haplotypes contain one copy of each autosome. This is standard practice. 

 Data has been properly deposited into INSDC databases and can be accessed. 

 In my opinion, this genome report is good, and no clarifications are needed. I would just suggest a revision of the abstract section which just seems to be the species taxonomy. An abstract with more details should be added.

Are sufficient details of methods and materials provided to allow replication by others?

Yes

Is the rationale for creating the dataset(s) clearly described?

Yes

Are the datasets clearly presented in a useable and accessible format?

Yes

Are the protocols appropriate and is the work technically sound?

Yes

Reviewer Expertise:

Genome assembly and annotation.

I confirm that I have read this submission and believe that I have an appropriate level of expertise to confirm that it is of an acceptable scientific standard.

# Reviewer report: Reviewer response for version 1

**DOI:** 10.21956/wellcomeopenres.26634.r123353

**Published:** 2025-05-29

**Authors:** Ranjana Bhaskar

**Affiliations:** 1 Zoological Survey of India, Southern Regional Centre, Santhome High Road, chennai, Tamilandu, India

The author performed the genome sequencing of the Ibiza wall lizard, *Podarcis* *pityusensis.* Sequencing was conducted using the PacBio platform, generating 35.44 Gb (gigabases) from 5.36 million reads. The genome assembly of *Podarcis* *pityusensis* was also carried out by the author. Assembly quality was assessed using BlobToolKit, and the snail plot provides an overview of assembly metrics and BUSCO gene completeness. The author also conducted the quality assessment.

 This study represents a novel addition to genomic databases and will allow for comparative analysis across the three species of the Western Islands group of *Podarcis*. The generated sequences will support ongoing conservation efforts and facilitate further research on population history, gene flow, and the drivers of phenotypic diversification within this species.

 I recommend this study for indexing.

Are sufficient details of methods and materials provided to allow replication by others?

Yes

Is the rationale for creating the dataset(s) clearly described?

Yes

Are the datasets clearly presented in a useable and accessible format?

Yes

Are the protocols appropriate and is the work technically sound?

Yes

Reviewer Expertise:

My area is conservation genetics, population genetics, Forensic science

I confirm that I have read this submission and believe that I have an appropriate level of expertise to confirm that it is of an acceptable scientific standard.

# Reviewer report: Reviewer response for version 1

**DOI:** 10.21956/wellcomeopenres.26634.r123793

**Published:** 2025-05-29

**Authors:** Hardip R. Patel, Paul Waters, Arthur Georges

**Affiliations:** 1 Australian National University, Canberra, Canberra, Australia; 2 University of New South Wales Science, Sydney, New South Wales, Australia

Review Prepared by Arthur Georges, Paul Waters and Hardip Patel

 We found this genome note to be technically sound but found the communication to be wanting. Part of this deficiency arises from lack of clarity on what the genome note is designed to achieve. A genome note is an announcement of the availability of a new genome assembly made available concurrently with the assembly file becoming available in a public repository. In this the genome note succeeds. However, it should also describe the sequences and how they were generated and analysed in sufficient detail for one to be able to repeat the workflow or, if necessary, turn to the genome note for insight as to why subsequent use of the genome assembly has delivered unexpected results. It should highlight any novel approaches to the assembly for the benefit of others. In this the genome note does not succeed. More detail is required as in for example the description of the progressive gains from the hifiasm base assembly, the value add of the YAHS scaffolding, and then the manual curation required and the value add of that manual curation. Finally, the genome note should point to knowledge gaps where this assembly can be used to advance our knowledge and understanding. Pointers to the future.

 We would like to see the genome note revised to bring out these sentient points. Otherwise, the value of the publication is not a lot greater than simply the submission of the assembly on NCBI.

**Abstract**

 The abstract needs to be included. Among other details expected in abstract, it should also include (a) a brief sentence or two introducing the species, its karyotype and why it is important; (b) “a female ZW specimen” so that one can see immediately that the heterogametic sex has been sequenced; (c) a statement of the key benchmark statistics for the assembly against the standard set published by the Earth BioGenome Project; (d) a statement to the effect that the assembly is published without annotation; and (e) a final concluding sentence or two on the value of having this assembly out. At the moment it contains species taxonomy only.

**Species Taxonomy**

 The NCBI abbreviated version would be more appropriate here. Eukaryota; Metazoa; Chordata; Craniata; Vertebrata; Euteleostomi; Lepidosauria; Squamata; Bifurcata; Unidentata; Episquamata; Laterata; Lacertibaenia; Lacertidae; *Podarcis* *pityusensis* (Boscá 1883) [NCBI:txid74359]

**Background**

 Notwithstanding the information being included in the abstract, the background needs to include a description of the karyotype and state clearly that the species has a ZZ/ZW system of sex determination, and that a female (ZW) animal has been sequenced.

 The authors could consider being more specific in how the generation of this assembly will provide the basis for an acceleration of new knowledge in conservation and comparative studies, and provide opportunity to address challenges that would otherwise be intractable.

**Genome Sequence Report**

 Consider using read depth in place of coverage, as the latter is unambiguous [throughout].

 Approximately 23.0x? Do you mean approximately 23x? And in any case 35.44Gb/1.46154 gives 24.25x. 35.44Gb output from a Revio SMRT Cell seems relatively low. Details on the number of SMRT cells and how they were used can inform other readers about expectation from a sequencing platform.

 As outlined earlier, it would be nice to see how the assembly progressed in terms of summary statistics from the base hifiasm assembly, to the YAHS assembly, to the final manual curated assembly. There are some details provided but not clear how different contigs/scaffolds progressed in the workflow. More detail is required on what the manual curation involved.

 What do you mean exactly by curated to chromosome level. Need to specify what you mean by chromosome level.

 It is not clear what the final assembly is. Is it Haplotype 1? Why not the hifiasm pseudohaplotype? Have the Z and the W been added to this final assembly? How? There is a discrepancy between the length of the final assembly and the genomescope estimate. This warrants a comment.

 The authors report the percentage of the assembly mapped to chromosomes, but the assembly has not been physically mapped. Should these be referred to as putative chromosomes?

 Most of the assembly sequence (99.0%) was assigned to 20 chromosomal-level scaffolds, representing 18 autosomes and the W and Z sex chromosomes. Is this consistent with the karyotype? Be sure to add citations for the karyotype.

 Not really enamored with Figure 2. This information might be better presented in a table. “A light grey spiral at the centre shows the cumulative scaffold count on a logarithmic scale” – not sure what this means. Where is the log scale? Very hard to interpret.

 Figure 3. I really struggled to see the different colours for the smaller dots in this graph. Suggest using more definitive colours.

 The paragraph immediately following Figure 3 needs a substantial rewrite.

 “The *k*-mer recovery for haplotype 1 is 86.42%, and for haplotype 2 82.68%, while the combined haplotypes have a *k*-mer recovery of 97.94%.” This likely arises from the high heterozygosity. Inclusion of a GenomeScope figure would assist the reader here. 97.94 recovery of k-mers seems lower as well.

**Methods**

 We were disappointed to see that the specimen used for the assembly was released. It should have been vouchered and lodged with a reputable museum. Standard morphometric measurements were taken but not reported in the manuscript.

 An assembly often includes mitochondria sequences as multiple contigs. Were they removed? More clarity on mitohifi would help here. Was mitohifi run on the reads or the assembly?

 “Detailed protocols are available on protocols.io (Denton *et al.*, 2023b)” – a quick perusal of these two papers shows this claim is not true. Need to add the quantity and type of the lysis buffer, quantity of the tissued used after dissection, etc. The specific protocol within these papers needs to be identified. Also confused as to how tissue 20-50 mg was used for the HiC when the lizard was released. Was the HiC from a different individual from the focal assembly individual?

 Inclusion of FemtoPulse results could inform other users working with similar samples to compare their DNA quality and assembly results.

 20-50 mg of frozen tissue? But only one individual. Which was it, 20 mg or 50 mg?

 “At a minimum, samples were required to have an average fragment size exceeding 8 kb and a total mass over 400 ng to proceed to the low input SMRTbell Prep Kit 3.0 protocol (Pacific Biosciences, California, USA), depending on genome size and sequencing depth required”. This is a generic statement presumably taken from the manufacturers guidelines. What did you actually do? Needs to be specific to the current study.

 “Samples were sequenced on Revio 25M SMRT cells”. How many?

 Hi-C wet lab experiments suggest multiple samples were processed at the same time (“ *in a 96-well plate format”*). Indicate this clearly. Sample contaminations are often an issue, and readers can benefit by referring to this detail if needed.

 Bioinformatics methods require parameters used to conduct analysis. If defaults were used, state that explicitly.

 “The assembly was decontaminated using the Assembly Screen for Cobionts and Contaminants (ASCC) pipeline.” Results of this would be useful to readers.

 “Any identified contamination, missed joins, and mis-joins were amended, and duplicate sequences were tagged and removed”. Can you provide details of how these decisions were made. For example, how is a mis-join identified?

 “The Hi-C reads were aligned using bwa-mem2”. The parameters and any additional details would be useful here.

 “The Hi-C alignments were converted into a contact map using BEDTools” Further details required here for reproducibility.

 The blobkit pipeline – what did you use it for. There is a statement on what it can be used for, but what did you specifically use it for in this assembly?

 “The genome will be annotated using available RNA-Seq data and presented through the Ensembl pipeline at the European Bioinformatics Institute”. Hmmm. Why no annotation here? What RNAseq data are available for this Endangered species? Greatly limits the utility of the assembly and thus the resource note.

Are sufficient details of methods and materials provided to allow replication by others?

Partly

Is the rationale for creating the dataset(s) clearly described?

Yes

Are the datasets clearly presented in a useable and accessible format?

Yes

Are the protocols appropriate and is the work technically sound?

Partly

Reviewer Expertise:

Comparative Genomics, Bioinformatics

We confirm that we have read this submission and believe that we have an appropriate level of expertise to confirm that it is of an acceptable scientific standard, however we have significant reservations, as outlined above.
